# Epigallocatechin-3-Gallate Plus Omega-3 Restores the Mitochondrial Complex I and F_0_F_1_-ATP Synthase Activities in PBMCs of Young Children with Down Syndrome: A Pilot Study of Safety and Efficacy

**DOI:** 10.3390/antiox10030469

**Published:** 2021-03-16

**Authors:** Iris Scala, Daniela Valenti, Valentina Scotto D’Aniello, Maria Marino, Maria Pia Riccio, Carmela Bravaccio, Rosa Anna Vacca, Pietro Strisciuglio

**Affiliations:** 1 Department of Maternal and Child Health, Federico II University Hospital, 80131 Naples, Italy; piariccio@gmail.com (M.P.R.); carmela.bravaccio@unina.it (C.B.); 2 Institute of Biomembranes, Bioenergetics and Molecular Biotechnologies, National Council of Research, 70126 Bari, Italy; d.valenti@ibiom.cnr.it; 3 Department of Translational Medical Sciences, Section of Pediatrics, Federico II University, 80131 Naples, Italy; valentinask8@alice.it (V.S.D.); mr.marino@alice.it (M.M.); pietro.strisciuglio@unina.it (P.S.)

**Keywords:** nutraceutical supplementation, Down syndrome, mitochondrial dysfunction, EGCG, developmental profiling, clinical study, complex I, complex V, ATP synthase, GMDS-ER

## Abstract

Down syndrome (DS) is a major genetic cause of intellectual disability. DS pathogenesis has not been fully elucidated, and no specific pharmacological therapy is available. DYRK1A overexpression, oxidative stress and mitochondrial dysfunction were described in trisomy 21. Epigallocatechin-3-gallate (EGCG) is a multimodal nutraceutical with antioxidant properties. EGCG inhibits DYRK1A overexpression and corrects DS mitochondrial dysfunction in vitro. The present study explores safety profiles in DS children aged 1–8 years treated with EGCG (10 mg/kg/die, suspended in omega-3, per os, in fasting conditions, for 6 months) and EGCG efficacy in restoring mitochondrial complex I and F_0_F_1_-ATP synthase (complex V) deficiency, assessed on PBMCs. The Griffiths Mental Developmental Scales—Extended Revised (GMDS-ER) was used for developmental profiling. Results show that decaffeinated EGCG (>90%) plus omega-3 is safe in DS children and effective in reverting the deficit of mitochondrial complex I and V activities. Decline of plasma folates was observed in 21% of EGCG-treated patients and should be carefully monitored. GMDS-ER scores did not show differences between the treated group compared to the DS control group. In conclusion, EGCG plus omega-3 can be safely administered under medical supervision in DS children aged 1–8 years to normalize mitochondria respiratory chain complex activities, while results on the improvement of developmental performance are still inconclusive.

## 1. Introduction

Down syndrome (DS), or trisomy 21 (T21), is a common chromosomal aneuploidy and a major genetic cause of developmental delay and intellectual disability. Besides a characteristic set of facial and physical features, DS is also characterized by hypotonia, congenital malformations, increased risk of leukemia, immune and endocrine abnormalities, and Alzheimer’s-like dementia [1,2].

DS pathogenesis remains elusive. The dual-specificity tyrosine phosphorylation regulated kinase 1A (DYRK1A) gene, mapping on chromosome 21 and hyper-expressed in all T21 tissues so far analyzed, is regarded as one of the principal culprits of DS clinical features [3,4]. DYRK1A phosphorylates several transcription factors, such as cAMP response element-binding protein (CREB) and nuclear factor of activated T-cells (NFAT), endocytic complex proteins, and AD-linked gene products. DYRK1A interacts with the regulator of calcineurin 1 (RCAN1), an endogenous inhibitor of calcineurin A, mapping on the DS critical region and overexpressed as well. Both genes contribute to the learning and memory deficit, altered synaptic plasticity, impaired cell cycle regulation, and AD-like neuropathology in DS animal models [3].

It is also known that oxidative stress (OS) and mitochondrial dysfunction play a key role in DS pathogenesis. Individuals with T21 display a pro-oxidant status [5], starting as early as the fetal period [6]. Cellular redox homeostasis is important for synaptic plasticity, neuroinflammation, vesicle-mediated transport, signal transduction, protein folding and degradation [7]. Membrane lipid peroxidation and protein deamidation have been demonstrated in DS cells as a consequence of a chronic oxidative microenvironment [8,9]. Redox imbalance is caused by both the enhanced production of reactive oxygen species (ROS) and the inhibition of antioxidant defense mechanisms [10,11]. Different genes mapping on chromosome 21 can promote ROS production, such as the superoxide dismutase 1 (SOD1), RCAN1 [12], and the amyloid-beta precursor protein (APP) genes [13,14]. Oxidative imbalance and mitochondrial dysfunction are tightly interconnected in DS. Most of chromosome 21 hyper-expressed genes that directly enhance OS also cause mitochondrial bioenergetics dysfunction through the down-regulation of signaling pathways controlling the mitochondrial function, including AMP-activated protein kinase (AMPK), cAMP/protein kinase A (PKA), peroxisome proliferator-activated receptor-γ coactivator l alpha (PGC-1α) and sirtuin 1 (Sirt1). This causes the reduction of the mitochondrial respiratory chain (MRC) complex I and of the F_0_F_1_-ATP synthase (complex V) activities as well as an oxidative phosphorylation deficit [15]. In turn, damaged mitochondria, in particular the dysfunctional MRC complex I, overproduce ROS [16] that T21 cells are unable to scavenge, with further increase of OS and oxidative damage [15]. In addition, T21 alters mitochondrial biogenesis, structure, dynamics, and metabolism [17,18]. The DS mitochondrial phenotype is also associated with neurogenesis impairment [18,19]. Indeed, it is well-known that mitochondria energy metabolism is crucial for the correct functioning of high-energy requiring cells, such as neurons, and strongly influences the distinct developmental steps of adult neurogenesis [1,20]. Therefore, mitochondria might be considered as primary or secondary targets in pharmacotherapy development for DS [15].

Several bioactive natural compounds modulate mitochondrial function [21]; among these, epigallocatechin-3-gallate (EGCG) is the most studied so far. EGCG is a member of the flavonoid polyphenol family, found in large amounts in green tea leaves, with strong antioxidant properties [21]. It has been proposed as dietary supplement in various disorders for its anticarcinogenic [22], anti-inflammatory [23], immunomodulatory and neuroprotective effects [24,25]. Extensive studies from our and other research groups on mice and cellular models have shown that EGCG can target mitochondria, producing a number of beneficial effects. These include ROS scavenging, regulation of mitochondrial homoeostasis [26], protection against β-amyloid-induced mitochondrial apoptosis [27], prevention of mitochondrial degeneration in aged rat brain [28], and modulation of mitochondrial biogenesis and bioenergetics [1,15]. In particular, EGCG activates the MRC complex I in fibroblasts and lymphoblastoid cells from DS subjects by modulating the cAMP/PKA signaling, which enhances the PKA-dependent phosphorylation of the complex I NDUFS4 subunit and its activity [29]. In neural hippocampal progenitor cells from the Ts65Dn mice, we also reported the EGCG-dependent activation of the AMPK/Sirt1/PGC-1α axis, which correlates with enhanced mitochondrial biogenesis and oxidative phosphorylation, as well as with the rescue of hippocampal neurogenesis [17]. EGCG is also a specific and safe inhibitor of DYRK1A [30] and it was found to improve brain defects induced by DYRK1A overexpression [31,32].

EGCG has been proposed as a nutraceutical candidate for the treatment of some DS phenotypic features [21,33]. So far, it was never tested in early infancy, when it could induce more positive effects [34]. EGCG has been investigated in two studies on adolescents and young adults with DS. In the first phase I study, EGCG (9 mg/kg per day; range 6.9–12.7) supplementation improved episodic and working memory with a good safety profile [32]. The following phase II trial showed that EGCG supplementation, associated with cognitive training, might improve visual recognition memory, inhibitory control, and adaptive behavior in young adults with DS [35]. Finally, a 6 month-treatment of a 10-year-old DS child with a combination of EGCG and fish oil omega-3, formerly shown to improve EGCG bioavailability [36], reverted mitochondrial dysfunction and improved attention skills [37]. The present pilot study explores safety profiles in young children with DS aged 1 to 8 years and the efficacy of EGCG in restoring mitochondrial complex I and V activity deficiency. A developmental profile has been also defined by means of the Griffiths Mental Developmental Scales—Extended Revised (GMDS-ER) before starting EGCG and after 6 months of therapy.

## 2. Subjects and Methods

### 2.1. Subjects

All subjects were recruited at the Genetic Unit of the Department of Maternal and Child Health, Federico II University Hospital, a referral Centre for Down syndrome. The study was conducted according to the guidelines of the Declaration of Helsinki and approved by the local Ethics Committee. Informed consent was obtained for all subjects involved in the study. Inclusion criteria for EGCG therapy were children aged 12 months to 8 years with diagnosis of T21 confirmed by karyotype (Group 1: DS-EGCG). Exclusion criteria were neurological disorders other than those expected for DS phenotype, and concomitant medical conditions, such as uncontrolled thyroid dysfunction, gastrointestinal, hepatic or kidney disorders that could alter the adsorption or metabolism of EGCG.

For data analysis, we took advantage of two different control groups.

The first group of controls (Group 2: DS-CT) was used for the analysis of the results of the GMDS-ER. Comparison between DS-EGCG and DS-CT groups was helpful to distinguish between developmental improvements due to the spontaneous child’s evolution with growth and the effects of EGCG. The DS-CT group was composed of DS children with full trisomy 21, who were not on EGCG nor omega-3 supplementation. This group performed only the GMDS-ER at T0 and T6. Group 2 was comparable to the DS-EGCG group for chronological age, motor and cognitive therapies (physiokinesis, psychomotor and language stimulation) and general quotient at enrollment, expressed in months of mental age (10 subjects, 5M/5F, mean chronological age at enrollment 37 ± 22 months; mental age 20 ± 5 months).

**Scheme 1:**
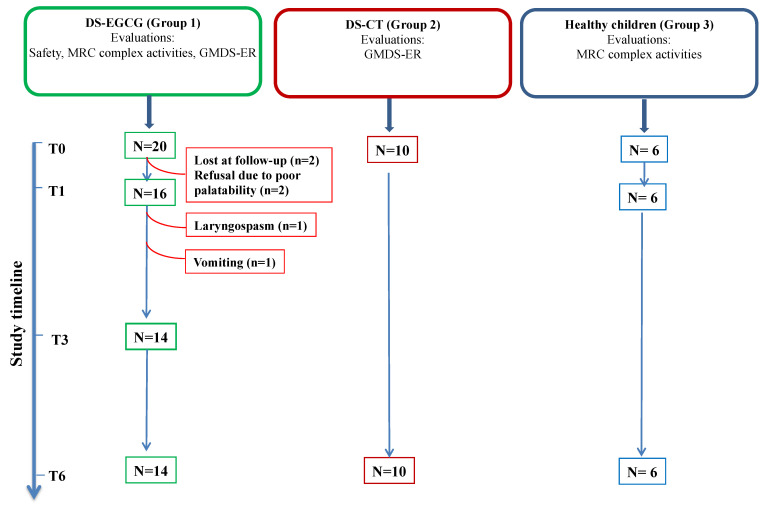
Study diagram. DS-EGCG: DS children treated with EGCG (Group 1); DS-CT: DS control group (Group 2); Healthy children (Group 3). N, patient number; safety evaluation was performed at baseline (T0), after 1 month (T1), after 3 months (T3) and after 6 months (T6) of EGCG supplementation. Griffiths Mental Developmental Scales—Extended Revised (GMDS-ER) evaluations were performed at T0 and at T6. Mitochondrial respiratory chain (MRC) complex activities were evaluated at T0, T1 and T6. Red lines indicate patient’s dropout.

The second control group (Group 3: Healthy children) was necessary to compare the measurement of MRC complex activities. It was composed of 6 healthy children without trisomy 21 (3F/3M, mean age 4 years, range 2–8 years) who came at the hospital for disorders other than DS, for which a normal mitochondrial function is expected (i.e congenital hypothyroidism, well controlled by L-tyroxine therapy) (Scheme 1).

The placebo group is not present in this study due to the unavailability of an inactive powder with the same taste and physical characteristics of pure EGCG and suitable for the use in young children who are unable to swallow capsules.

#### 2.1.1. Study Endpoints

The primary endpoints were (i) the evaluation of safety and tolerability of EGCG in young children with DS and (ii) the assessment of EGCG efficacy in improving mitochondrial respiratory chain complex I and V activities in peripheral blood mononuclear cells (PBMCs) of DS children. The improvement of at least one of the GMDS-ER (0–8 years) subscales was considered as a secondary endpoint.

#### 2.1.2. EGCG Supplementation

Pure caffeine-free EGCG (EGCG > 90%, powder) was prescribed at a dosage of 10 mg/kg/day, once daily, per os, for 6 months. The dose calculated for each child according to body-weight was specifically incapsulated in pharmacy starting from the powder. To prevent oxidation, parents were instructed to open the capsule before administration and to suspend the powder in omega-3 (half or one teaspoon depending on the amount of EGCG: 250–500 mg EPA + DHA/day) to enhances EGCG bioavailability, as previously described [37]. Ascorbic acid also improves EGCG bioavailability by preventing oxidation [38] and was used as a second-line delivery product if omega-3 was not accepted due to poor palatability. Indeed, EGCG is labile in aqueous solutions due to auto-oxidization and epimerization. Ascorbic acid was used only in one patient who stopped EGCG at the second month of supplementation. All other children suspended EGCG in omega-3. Finally, to avoid interaction with food and iron chelation, parents were asked to administer the product in fasting conditions.

#### 2.1.3. Visit Schedule

Visits were performed at T0 (before starting EGCG), at T1 (after 1-month of supplementation), at T3 (after 3-months of supplementation) and T6 (after 6-months of supplementation) (Table 1). Visits included anamnestic records, pediatric evaluation, biochemistry (complete blood count, ferritin, AST, ALT, GGT, creatinine, folic acid, homocysteine, thyroid function, screening for coeliac disease), mitochondrial complex activities and the Griffiths Mental Developmental Scales—Extended Revised (GMDS-ER).

#### 2.1.4. Withdrawal Criteria

EGCG has no cumulative effects; its half-life is 3.4 ± 0.3 h [39]. Therefore, EGCG can be withdrawn without concerns. Families were informed that they could withdraw consent and stop EGCG administration whenever they wanted. EGCG was withdrew upon family request or upon medical judgement.

### 2.2. Measurement of MRC Complex Activities in Peripheral Blood Mononuclear Cells

PBMCs (0.5–1 × 10^6^ cells) were recovered from 5 mL peripheral whole blood through Percoll density gradient, washed with PBS, frozen in liquid nitrogen, and kept at −80 °C until later use. Measurements of MRC complex I (NADH:ubiquinone oxidoreductase), complex II (succinate:ubiquinone oxidoreductase), complex III (cytochrome *c* oxidoreductase), complex IV (cytochrome *c* oxidase) and complex V (ATP synthase) activities were carried out in mitochondrial membrane-enriched fractions obtained from frozen lymphocytes thawed at 2–4 °C, suspended in 1 mL of 10 mM Tris-HCl (pH 7.5), supplemented with 1 mg/mL BSA, and exposed to ultrasound energy for 8 sec at 0 °C (11 pulse 0.7 sec on, 0.7 sec off) at 20 kHz, intensity 2. The ultrasound-treated cells were centrifuged (10 min at 600× *g*, 4 °C). The supernatant was collected and centrifuged again (10 min at 14,000× *g*, 4 °C) to obtain a mitochondrial pellet, that was suspended in 0.2 mL of the respiratory medium consisting of 210 mM mannitol, 70 mM sucrose, 20 mM Tris/HCl, 5 mM KH_2_PO_4_/K_2_HPO_4_, (pH 7.4) plus 5 mg/mL BSA, 3 mM MgCl_2_.

Measurement of MRC complex activities were performed as previously described [37,40] by three assays relying on the sequential addition of reagents to measure: (i) the rotenone-sensitive complex I (as NADH decylubiquinone reductase activity) followed by the oligomycin-sensitive ATPase activity of complex V; (ii) malonate-sensitive complex II activity; (iii) the activity of complex IV trigged by the reduced cytochrome *c* followed by the antimycin-sensitive complex III. MRC complex specific activities (nmoles/min/mg of mitochondrial proteins) were measured in the DS treated group and reported as % of the control (non trisomic PBMCs) MRC complex activities.

### 2.3. Griffiths Mental Developmental Scales—Extended Revised

The GMDS-ER was published in 2006, updating the 1996 version [41,42]. It consists of 2 sets of scales, one for each age-group: 0–2 years and 2–8 years. The GMDS-ER includes 6 sub-scales: Locomotor (subscale A), Personal-Social (subscale B), Language (subscale C), Eye and Hand Co-ordination (subscale D), Performance (subscale E), and Practical Reasoning (subscale F). The latter subscale applies only to children aged 2 to 8 years. Raw scores were converted into mental age scores, expressed in months for each developmental area.

### 2.4. Statistical Analysis

The normality of continuous data was assessed by the Shapiro–Wilk test. Related continuous data were analyzed with the Student’s *t*-test or the Wilcoxon test in the case of normal or not-normal distribution of the data set, respectively. Mann–Whitney was used in the case of not-normal distribution of unrelated datasets. A two-tailed *p*-value < 0.05 was considered as statistically significant.

## 3. Results

### 3.1. Patients’ Diagram and Demographics

Twenty DS subjects with full trisomy 21 (11 males/9 females) were enrolled in this pilot study on EGCG supplementation. The mean age at enrollment was 44 ± 27 months (age range 1–8 years).

Of these 20 subjects, 6 were unable to complete the study protocol due to the following reasons: two patients were lost at follow-up; two patients refused EGCG due to poor palatability; one patient presented laryngospasm after 1 month of supplementation and stopped EGCG; one patient presented persisting vomiting starting from the 2nd month of supplementation and EGCG was withdrawn (see also Safety and Tolerability section). Hence, 14 DS children completed the study (Scheme 1) and were included in the data analysis. Of these patients, 8 were males (57%) and 6 (43%) were females. The mean chronological age at enrollment was 41 ± 26 months (age range 1–8 years) and the mean mental age at enrollment was 22 ± 12 months (Table 2). A control group of 10 DS subjects with full trisomy 21 with comparable chronological age, mental age and medical comorbidities at enrollment (Group 2) was used to interpret the results obtained by the GMDS-ER (Table 2).

### 3.2. Safety and Tolerability of EGCG Supplementation

The biochemical data and their variations, analyzed at the baseline and after 1 and 6 months of EGCG supplementation (T0, T1, T6), are shown in Table 3. Folic acid was dosed also after 3 months of nutraceutical supplementation (T3). No significant variations of hepatic, renal, or thyroid function were noted in DS children considered both individually and as a group, nor decrease of ferritin status. Lipid profile did not show any significant difference throughout the study.

**Figure 1:**
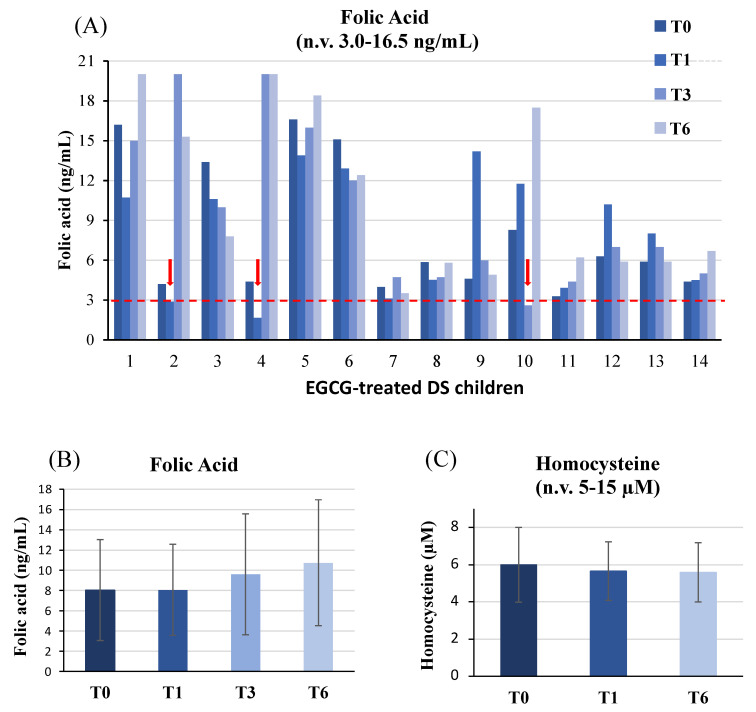
Plasma folate and homocysteine levels. Folate levels analyzed in EGCG-treated DS children (**A**) and the mean values of plasma folic acid (**B**) and homocysteine (**C**) levels are depicted. In panels 1B and 1C, mean values at individual time-points are not significantly different (*p* > 0.05) compared to baseline (T0).

Mean plasma folate level in DS children, considered as a group, did not show significant differences during the study (Figure 1B). However, when patients’ profiles were analyzed individually, plasma folic acid decreased below the lower reference range in 3 out of 14 DS children (21.4%) as shown in Figure 1A. In patient #2, folic acid dropped from 4.2 ng/mL at the baseline to 2.9 ng/mL after 1 month of supplementation (T1); in patient #4, folic acid declined from 4.4 ng/mL at T0 to 1.65 ng/mL at T1; in patient #10, folic acid lowered to 2.6 ng/mL after 3 months of EGCG (T3) supplementation. Those children were treated with levofolinic acid (2.5 mg on alternate days) for 2 months with restoration of plasma folates while keeping EGCG dose unchanged until T6. We did not observe any decline of folic acid after the end of levofolinic acid supplementation.

Homocysteine levels were also dosed in our cohort at T0, T1 and T6; no difference was detected during EGCG therapy compared to the baseline (Figure 1C).

EGCG was withdrawn in 2 patients after the first month of EGCG supplementation. The first child was a 3-year-old boy with no history of allergies. On day 33 from the baseline of EGCG + omega-3 therapy, he experienced laryngospasm requiring intra-muscular corticosteroid injection. He was treated at home by the primary care pediatrician, did not require hospitalization and fully recovered. The family refused a skin allergy patch test with both omega-3 and EGCG and we could not link the adverse event to the therapy. The family asked to stop EGCG. The second case concerned a 4-year-old girl who had been taking EGCG + omega-3 for one month without any medical concern. During the second month of treatment, she presented vomiting even after several hours from the assumption of EGCG. Vomiting persisted after the substitution of omega-3 with ascorbic acid, while it was absent when the daily EGCG dose was omitted. This adverse effect was assumed to be linked to EGCG. Once the treatment was withdrawn by physicians, the symptom resolved without sequelae.

### 3.3. Efficacy of EGCG Supplementation in Restoring MRC Complex I and V Activity

**Figure 2:**
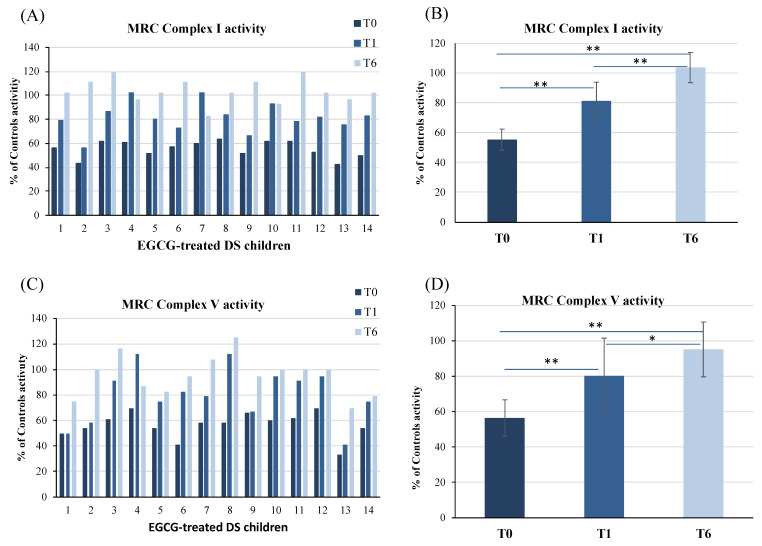
Mitochondrial respiratory chain complex I and complex V activities in PBMCs of DS children before (T0), after 1 (T1) and 6 (T6) months of nutraceutical supplementation with EGCG. Complex I and V activities are expressed as % of controls activity. Controls were 6 age-matched children without trisomy 21 (Group 3). Data reported in (**A**,**C**) are the mean value of two different measurements. MRC Complex I and V activities mean values of all EGCG-treated DS patients are reported in panels (**B**,**D**). Bars represent mean ± SD. * *p* < 0.05, ** *p* < 0.001.

The mitochondrial complex I and V activities were measured in PBMCs of DS children before EGCG supplementation (T0) and after 1 (T1) and 6 months (T6) of therapy (Figure 2A,C). Results are represented as percentage of the MRC complex activity of the control group without T21 (Group 3, healthy children).

We previously demonstrated that aberrant mitochondrial energy metabolism in peripheral cells, such as fibroblasts, lymphoblastoid cells [29] and PBMCs [37], is characterized by a reduction of the MRC I and V complexes and normal MRC II, III and IV activities. In DS treated with EGCG, we confirmed that at the baseline there was a significant reduction of the activity of both complexes I and V. At T0, complex I and V activities of DS children were 55.5% (mean ± SD = 55.5 ± 7) and 56.5% of control subjects (mean ± SD = 56.5 ± 10), respectively (Figure 2B,D).

During EGCG supplementation, a progressive increase of complex I and V activities occurred in all patients (Figure 2A,C). In the DS treated group, mean complex I and V activities fully restored at T6 (Figure 2B,D). In detail, complex I activity raised to 81.5% (mean ± SD = 81.5 ± 12.5) (*p* < 0.0001) at T1 and to 104% (mean ± SD = 104 ± 10) (*p* < 0.0001) at T6 (Figure 2B). Complex V activity also increased to 80% (mean ± SD = 80 ± 21) (*p* = 0.0008) at T1 and to 95% (mean ± SD = 95 ± 15) (*p* < 0.0001) at T6 (Figure 2D). No significant changes in the activities of complexes II, III and IV were found neither at the baseline nor during treatment (data not shown).

Taken together, results indicate that EGCG, delivered with omega-3, is effective in restoring MRC complex I and V activities in DS.

### 3.4. Effect of EGCG Supplementation on Developmental Performance Measured by the Griffiths Mental Developmental Scales—Extended Revised (GMDS-ER)

DS children belonging to the EGCG-treated and untreated groups (Groups 1 and 2) performed the GMDS-ER 0–2 or 2–8 years depending on their chronological age at enrollment. DS subjects were analyzed both as entire groups (1–8 y) (Groups 1 and 2) and subgroups of children aged ≤2 years (Groups 1a and 2a) or 2–8 years (Groups 1b and 2b). Intra-subject comparisons were performed between T0 and T6 scores (Table 4 and Table 5).

At the baseline, chronological age and General Quotient (GQ), expressed in months of mental age, were comparable in the group of treated and untreated DS subjects (Group 1 and 2) (*p* > 0.05). Therefore, patients of Group 1 and 2 had the same developmental level at enrollment. In addition, all patients carried-out rehabilitation therapy that was not modified during the observation period (T0–T6). In both groups, GQ scores highlighted that only in the subgroups aged ≤2 years the developmental acquisitions significantly improved after six months. On the contrary, the developmental progress in patients aged 2–8 years was not significant after six months, neither in group 1 nor 2.

These data appear consistent with the results of the individual GMDS-ER subscales. In fact, both groups 1 and 2 had similar significant improvements in the locomotor, personal-social, language and performance subscales. Mental age significantly improved in younger children aged 1–2 years of both the treated and untreated groups, while no significant progress was recorded in the subgroups aged 2–8 years, with the only exception of the Personal-Social subscale in the treated group (group 1b).

Results show that DS patients (both treated and untreated) improve developmental skills more rapidly in the first years of their lives. Acquisitions of psychomotor and cognitive skills slow-down with growth, so that improvements may be hardly detectable over a short time span. These results indicate that improvements in developmental performance observed in DS children treated with EGCG are independent of EGCG supplementation and are rather the expression of the spontaneous developmental age-related improvements observed in DS.

## 4. Discussion

Trisomy 21 is a multiorgan disorder characterized by muscular hypotonia, developmental delay and intellectual disability. Later in life, neurodegeneration begins with Alzheimer’s-like neuropathological lesions. Ever since adolescence, psychiatric disorders may occur in some individuals, impacting cognition and performance. Other comorbidities variably affect DS individuals, such as congenital malformations, eye disorders, hearing impairment, skeletal involvement, endocrinological and immune dysregulations. The triplication of chromosome 21 genes causes a widely downstream expression derangement of genes mapping on other chromosomes and a cascade impairment of a myriad of pathways and biochemical processes [43,44]. Hence, the pathogenesis of DS remains largely elusive and no specific therapy has been developed for the treatment of its neurobiological dysfunctions. An additional challenge in developing pharmacological therapies is the lack of definitive evidence that the currently used DS mice models adequately mimic human DS. In fact, translational clinical trials are still few and heterogeneous regarding the investigated experimental molecules and the subjects’ age at enrollment. Experimental products indicated for the pediatric age should be primarily selected to improve neurocognitive prognosis. In parallel, while unravelling the complex network of gene-gene interactions, the discovery of individual biochemical functions impaired in trisomy 21 may help to find active substances able to revert a biochemical phenotype. In the last years, DYRK1A overexpression, oxidative stress and mitochondrial dysfunction have been described as contributors to the pathogenesis of DS.

In this scenario, EGCG is a multimodal nutraceutical able to intercept some of the DS biochemical dysfunctions. EGCG can cross the brain-blood [24] and the placental [45] barriers, is a powerful antioxidant, prevents oxidative phosphorylation deficit, promotes mitochondrial biogenesis and inhibits DYRK1A hyperactivity [1,15,21]. In DS, EGCG has been currently used in adolescents and adults [32,35], but data on the safety in DS children are lacking. In the present study, we explored the safety of EGCG in young children with DS and its efficacy in restoring MRC complex I and V activities. Results show that EGCG is well tolerated in children aged 1–8 years treated for 6 months. No significant variation of hepatic, renal, or thyroid function was noted in DS children considered individually or as a group, nor decrease of ferritin status. No serious adverse event was registered. Nonetheless, some issues should be underlined. Being young children unable to swallow capsules, poor palatability of EGCG may be challenging. EGCG also caused vomiting in one patient during the second month of supplementation and consequent study drop-out. Literature data suggested that plasma folates could decline during EGCG, putatively due to competitive inhibition of the DHFR enzyme [45]; indeed, reduction of plasma folates was observed in 3/14 (21.4%) of the subjects who completed the study. Those children were supplemented with levofolinic acid for two months with restoration of plasma folate levels that persisted within the normal range after the end of the treatment. In all 3 subjects, folic acid declined in the first 3 months of EGCG therapy, suggesting the need of a close follow-up during the first months of supplementation. Accordingly, the reduction of plasma folates was also described after the first month of supplementation in a 10-year-old boy treated with EGCG [37], while plasma folates had not been dosed in the two previous clinical studies on DS adolescents and adults [32,35]. One 3-year-old boy presented laryngospasm after one month of EGCG + omega-3. An history of allergy was not reported in this patient. Laryngospasm is very frequently observed in the pediatric age; this child did not require hospitalization and recovered without sequelae. In this case, we were unable to establish a relation between EGCG + omega-3 and laryngospasm.

As EGCG is a natural compound, families often purchase this green tea extract from the market and use it in self-medication for their children without medical supervision. Indeed, a recent study showed that parents of DS children often administer supplements starting from the first months of life, and sometimes from the prenatal period, without medical advice and unaware of the potential side effects [46]. Our experience underlines the importance of medical supervision and monitoring during EGCG supplementation. Medical supervision is not only necessary to prevent/identify possible side effects, but also to detect clinical benefits. It is indeed important to give families the correct information on harms but also expected benefits of a therapy. Medical supervision is also crucial to avoid products that contain multiple polyphenols, whose effect is not sufficiently studied in DS, and to advice patients on the correct dosage for body-weight.

Regarding efficacy parameters, our study shows that pure EGCG (EGCG > 90%) delivered with omega-3 is effective in reverting the deficit of the respiratory chain complex I and V activities, crucial for mitochondrial energy production and prevention of OS. At the baseline, complex I and V activities were 55.5% and 56.5% of control samples, respectively. At the end of the study, both activities were restored. MRC complex I is a key target for ROS homeostasis and the main producer of ROS when dysfunctional [47]. Indeed, we previously demonstrated that the reduced catalytic activity of complex I is the primary cause of increased mitochondrial ROS in DS human peripheral cells [16]. Thus, the rescue of complex I activity observed in this study is expected to prevent OS. Mitochondria are pivotal in energy production. Their function is particularly important in high energy requiring cells, such as liver, kidney, muscular and neuronal cells. Mitochondria play a central role in many other metabolic pathways, such as calcium signaling (including calcium-evoked apoptosis) [48], steroid synthesis [49], hormonal signaling [50] and immune signaling [51]. Mitochondrial estrogen receptors have been found in various tissues and cell types, including brain [52] and heart [53]. Mitochondrial dysfunctions occur during aging [5] and mitochondrial biogenesis, bioenergetics and dynamics play a central role in neurogenesis and neuroplasticity [1]. Consistently, dysfunctional mitochondria and altered redox homeostasis are involved not only in the pathogenesis of DS, but also in other neurodevelopmental disorders such as Rett syndrome, Fragile X syndrome, autism spectrum disorders, attention deficit hyperactivity disorder, for which targeting mitochondria is now considered as a therapeutic opportunity [54,55,56,57,58]. Therefore, the restoring of mitochondrial function obtained after EGCG supplementation is potentially relevant for DS phenotype.

Finally, as secondary endpoint, we decided to explore whether EGCG could improve mental and psychomotor development in DS children by means of the GMDS-ER. Regarding this endpoint, the study presents several limitations. The first one is the lack of a placebo group. This is an intrinsic problem when EGCG powder cannot be masked into capsules. In fact, EGCG has a characteristic bitter taste that is difficult to imitate with an inactive powder. In previous studies involving young DS adults [35], subjects of both the EGCG and the placebo groups were able to swallow capsules [35]; in a following trial, enrolling 6 to 12 years-old DS children, a 100 mL-flavored drink containing green tea extract or placebo was developed (ClinicalTrials.gov Identifier: NCT03624556). The latter drug delivery option was nonetheless arduous in very young DS children (1–8 years-old), as those involved in our study, mostly incapable of drinking quickly a 100 mL-mixture, taking into account the lability of EGCG in aqueous solutions [59]. Another study limitation is the small number of treated and untreated DS groups, especially when subgroups are considered (≤2 years; 2–8 years). Also, the comparison of mental development among children with cognitive impairment deals with a great range of variability such as rehabilitating therapies, family, school, and social environment. Finally, EGCG was administered with omega-3 used as a vehicle to improve bioavailability, but the effect of omega-3 alone was not studied, and literature data are still lacking. Considering the mentioned limitations, the present pilot study compares developmental profiles of DS subjects supplemented with EGCG to those obtained in a control group of DS children homogeneous with the treated group for chronological age, mental age, rehabilitation therapies, and medical co-morbidities. Results show that in the analyzed time-period, i.e., 6 months, young children with DS treated with EGCG, especially the group aged ≤2 years, improved their locomotor, personal-social, language and performance skills; however, the improvement is similar to that observed in the age-matched DS control group. Therefore, results indicate that improvements in developmental performance observed in DS children treated with EGCG are independent of EGCG supplementation and are rather the expression of the natural developmental age-related profile observed in DS. In our opinion, these negative data do not exclude that EGCG could impact developmental prognosis by means of its multimodal action on mitochondria, DYRK1A and oxidative stress; in fact, the absence of a significant difference in developmental skill improvements between DS-EGCG and DS-CT groups may also depend on the study design and observation period, possibly inadequate to detect those putative effects. Indeed, this study was primarily designed to achieve clues on the safety and tolerability of EGCG in young children with DS and to confirm in vivo the results on mitochondria function obtained in cell models.

Some questions remain to be solved such as the most appropriate mode of delivery, the quality of EGCG (green tea extracts or pure EGCG), the combination of EGCG with other nutraceuticals and the dose-response relationship. At this regard, the lack of advantageous therapeutic behavioral effects and potentially detrimental skeletal outcomes of high EGCG dose have been reported in Ts65Dn mice [60,61]. Another study showed that even using a pure stabilized EGCG in the concentrations producing therapeutic effects on skeletal phenotypes, EGCG failed to improve cognitive phenotypes in adolescent DS mice [62]. These reports and the present study emphasize the importance of EGCG dosage-effect relationship and necessity of medical supervision in polyphenol supplementation. It should also be considered that prolonged EGCG administration is likely to be necessary, given the loss in adulthood of the benefits of the neonatal EGCG treatment of Ts65Dn mice [63].

## 5. Conclusions

In the absence of alternative therapies to improve prognosis in DS subjects, we think that supplementation with decaffeinated pure EGCG at a controlled dose and under medical supervision should be encouraged in subjects with trisomy 21 with the aim of improving mitochondria energy production. Indeed, the restoration of such a crucial biochemical process by means of EGCG is expected to positively impact cell metabolism. Regarding cognitive improvements, parents should be informed that efficacy data are still lacking in young children with DS. Future clinical studies addressing the interaction between EGCG and omega-3 as well as the analysis of EGCG effects on other relevant targets such as DYRK1A, mtDNA and ROS defense proteins will expand the knowledge of drug efficacy for this orphan disease. Long term follow-up studies on large cohorts of DS children precociously treated, prospectively characterized in their cognitive and clinical phenotype, could better assess the impact of EGCG on the natural history of DS. To date, the developmental natural history of DS children cohorts, and its variability, has been only roughly traced. The depiction of developmental performance trajectories in large cohorts of young children with DS could help clinical researchers evaluate the effects of molecules candidate for the treatment of DS, especially when the production of a suitable placebo is challenging.

## Figures and Tables

**Table 1:** Study flow-chart for Down syndrome (DS) subjects treated with epigallocatechin-3-gallate (EGCG) for 6 months (Group 1).

Assesments	T0	T1	T3	T6
Anamnestic record	x	x	x	x
Pediatric evaluation	x	x	x	x
Complete blood count, ferritin, AST, ALT, GGT, LDH, urea, creatinine, TSH, FT3, FT4, anti-transglutaminase antibodies, homocysteine	x	x		x
Folic acid	x	x	x	x
Mitochondrial complex activities	x	x		x
GMDS-ER	x			x

Abbreviations: AST: aspartate aminotransferase, ALT: alanine aminotransferase, GGT: gamma-glutamyl transferase, LDH: lactate dehydrogenase, TSH: thyroid stimulating hormone, FT3: free triiodothyronine, FT4: free thyroxine, GMDS-ER: Griffiths Mental Developmental Scales—Extended Revised.

**Table 2:** Patients’ demographics at enrollment.

Patients’ Demographics	DS-EGCG (Group 1; *n* = 14)	DS-CT (Group 2; *n* = 10)
Male/Female (%)	57/43	50/50
Karyotype	Full Trisomy 21	Full Trisomy 21
Chronological age (months, M ± DS)	41 ± 26	37 ± 22
Patients aged ≤2 years (*n*)	7	5
Patients aged 2–8 years (*n*)	7	5
General quotient mental age (months, M ± DS)	22 ± 12	20 ± 5
Congenital Heart Disease	2/14	1/10
Hypothyroidism (on L-Tyroxine therapy)	2/14 *	1/10 *
Coeliac disease	0/14	0/10
Obstructive sleep apnea (OSAS)	2/14 ^§^	1/10 ^§^
Hypoacusia	0/14	0/10
Visual impairment	2/14 ^Ɨ^	2/10 °

DS-EGCG: DS children treated with EGCG; DS-CT: DS control group; * acquired hypothyroidism controlled by L-Tyroxine therapy; ^§^ mild OSAS diagnosed by polysomnography; ^Ɨ^ nystagmus; ° strabismus.

**Table 3:** Biochemical profiles of DS children treated with EGCG for 6 months (Group 1).

Biochemical Analysis.	T0	T1	T3	T6
Folic acid (n.v. 3–16.5 ng/mL)	8 ± 5	8.1 ± 4.6	9.6 ± 6	11 ± 6
Homocysteine (n.v. 5–15 µM)	6 ± 2	5.7 ± 1.6	n.p.	5.6 ±2
Ferritin (n.v. 5–100 ng/mL)	46 ± 37	45 ± 40	n.p.	40 ± 36
AST (n.v. <34 U/L)	37 ± 13	30 ± 7	n.p.	35 ± 6
ALT (n.v. <55 U/L)	20 ± 7	21 ± 6	n.p.	19 ± 5
LDH (n.v. 125–200 U/L)	345 ± 171	292 ± 79	n.p.	302 ± 63
GGT (n.v. 9–36 U/L)	20 ± 6	18 ± 5	n.p.	21 ± 8
Cholesterol (n.v. <190 mg/dL)	170 ± 15	165 ± 20	n.p.	168 ± 13
Triglycerides (n.v. <150 mg/dL)	100 ± 20	95 ± 18	n.p.	98 ± 26
Glucose (n.v. 60–100 mg/dL)	72 ± 9	73 ± 6	n.p.	69 ± 5
Creatinin (n.v. 0.6–1.1 mg/dL)	0.5 ± 0.05	0.5 ± 0.06	n.p.	0.5 ± 0.06
Urea (n.v. 15–36 mg/dL)	33 ± 9	34 ± 8	n.p.	32 ± 6
TSH (n.v. 0.64–6.27 µU/mL)	4.3 ± 1.7	4.4 ± 2	n.p.	4 ± 1.8
FT3 (n.v. 2–4.4 pg/mL)	4.3 ± 0.6	4.1 ± 0.8	n.p.	4.2 ± 0.5
FT4 n.v. 0.9–1.7 ng/dL)	1.3 ± 0.2	1.4 ± 0.3	n.p.	1.3 ± 0.9
Complete Blood Count	Normal	Normal	n.p.	Normal
Antitransglutaminase IgA	Negative	Negative	n.p.	Negative

The difference between T1, T3, T6 vs. T0 is not significant for all the analytes. Within the complete blood count, hemoglobin, white blood cells and platelets resulted in the normal range for all patients throughout the study. Data are expressed as mean ± SD; n.p. = not performed.

**Table 4:** Results of the GMDS-ER analysis in EGCG-treated DS children.

Items	DS-EGCG (1–8 y) (*n* = 14) Group 1		*p*-Value	DS-EGCG (≤ 2 y) (*n* = 7) Group 1a		*p*-Value	DS-EGCG (2–8 y) (*n* = 7) Group 1b		*p*-Value
	T0	T6	T6 vs. T0	T0	T6	T6 vs. T0	T0	T6	T6 vs. T0
Chronologica age (CA)	41 ± 26	47 ± 26	n.s	20 ± 4	26 ± 4	n.s	62.5 ± 20	69 ± 20	n.s
General Quotient—Age-Equivalent (AE)	22 ± 12	24 ± 11	n.s	14 ± 3	18 ± 3	0.05	29 ± 12	30 ± 12	n.s
Gap between chronological age and AE general quotient (CA-AE)	19 ± 16	23 ± 18	<0.01	6 ± 3	8 ± 4	n.s	33 ± 12	38 ± 12	n.s
Locomotor (subscale A)	21 ± 12	25 ± 14	<0.01	13 ± 4	16 ± 4	n.s	30 ± 12	35 ± 15	n.s
Personal-Social (subscale B)	24 ± 18	29 ± 18	<0.01	15 ± 4	19 ± 4	<0.05	33 ± 16	39 ± 21	<0.05
Language (subscale C)	20 ± 12	23 ± 15	<0.01	12 ± 2	15 ± 2	<0.05	27 ± 14	31 ± 19	n.s
Eye and Hand Co-ordination (subscale D)	24 ±14	27 ± 16	n.s	15 ± 5	18 ± 3	n.s	3S3 ± 14	36 ± 19	n.s
Performance (subscale E)	21 ± 9	23 ± 11	<0.01	16 ± 5	17 ± 3	n.s	27 ± 9	30 ± 17	n.s
Practical Reasoning (subscale F)	NA	NA	n.s	NA	NA	NA	30 ± 8	33 ± 11	n.s

DS, Down syndrome; NA, not applicable; n.s, not significant. All data are expressed in months (mean ± SD). Data have been analyzed as entire group and subgroups according to chronological age at enrolment ≤ 2 years and 2–8 years. Practical Reasoning (subscale F) is present only in the GMDS-ER 2–8 and applies to children aged 2–8 years.

**Table 5:** Results of the GMDS-ER analysis in untreated DS control group.

Items	DS-CT (1–8 y) (*n* = 10) Group 2		*p*-Value	DS-CT (≤ 2 y) (*n* = 5) Group 2a		*p*-Value	DS-CT (2–8 y) (*n* = 5) Group 2b		*p*-Value
	T0	T6	T6 vs. T0	T0	T6	T6 vs. T0	T0	T6	T6 vs. T0
Chronological age (CA)	37 ± 22	43 ± 22	n.s	18 ± 5	24 ± 5		56 ± 15	62 ± 15	n.s
General Quotient—Age-Equivalent (AE)	20 ± 5	24 ± 11	n.s	11± 3	16 ± 2	0.01	29 ± 10	33 ± 9	n.s
Gap between chronological age and AE general quotient (CA-AE)	17 ± 12	18 ± 12	n.s	7± 4	8 ± 4		27 ± 7	29 ± 7	n.s
Locomotor (subscale A)	22 ± 14	25 ±16	<0.01	10± 2	12 ± 2		33 ± 10	39 ± 11	n.s
Personal-Social (subscale B)	24 ± 12	28 ± 14	<0.01	16± 5	18 ± 4		33 ± 10	39 ± 11	n.s
Language (subscale C)	19 ± 11	21± 10	<0.01	11± 2	15 ± 1	0.02	26 ± 11	28 ± 11	n.s
Eye and Hand Co-ordination (subscale D)	24 ± 14	27 ± 12	n.s	13 ± 4	18 ± 4		34 ± 13	37 ± 10	n.s
Performance (subscale E)	21 ± 9	25 ± 8	<0.01	14 ± 4	20 ± 4	0.01	29 ± 7	31 ± 9	n.s
Practical Reasoning (subscale F)	NA	NA	n.s	NA	NA	NA	31 ± 9	31 ± 7	n.s

DS, Down syndrome; CT, controls; NA, not applicable; n.s, not significant. All data are expressed in months (mean ± SD). Data have been analyzed as entire group and subgroups according to chronological age at enrolment ≤ 2 years and 2–8 years. Practical Reasoning (subscale F) is present only in the GMDS-ER 2–8 and applies to children aged 2–8 years.

## Data Availability

The data presented in this study are fully available in the Results section.
